# Supplementary material for: Brain Transcriptomics Across Diverse Sleep-Wake Manipulations Reveals Multiple Homeostatic Pathways in Drosophila
Source: bioRxiv. 2026 Mar 3:2026.02.28.708752. Preprint. [Version 1] doi: 10.64898/2026.02.28.708752 (PMC13001341; doi:10.64898/2026.02.28.708752)
Supplement: Supplement 8 [file NIHPP2026.02.28.708752v1-supplement-8.pdf]

bioRxiv preprint doi: https://doi.org/10.64898/2026.02.28.708752; this version posted March 3, 2026. The copyright holder for this preprint (which was not certified by peer review) is the author/funder, who has granted bioRxiv a license to display the preprint in perpetuity. It is made available under aCC-BY 4.0 International license.

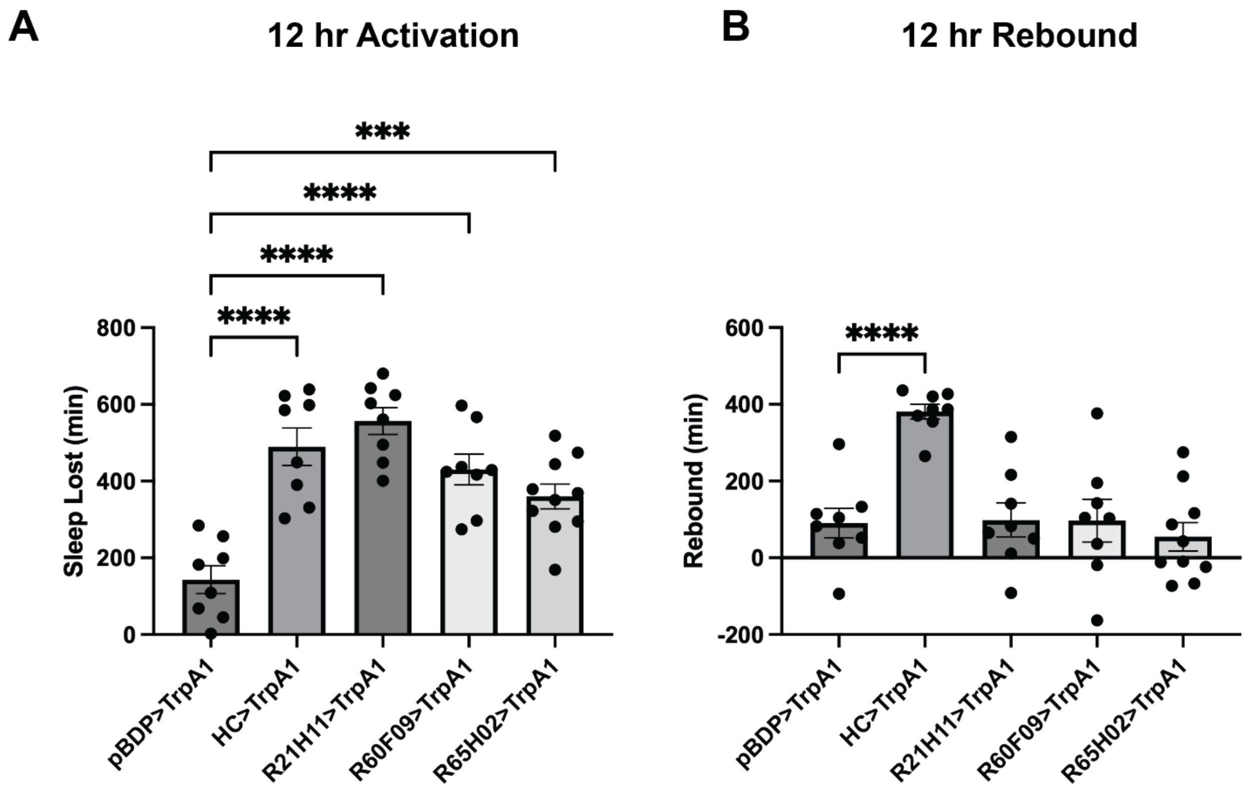

Figure S1 Wake Promotion and Rebound Sleep after 12h TrpA1 Activation

GAL4 lines for sleep deprivation were chosen following 12 hr activation experiments.

A. Sleep lost during 12 hr nighttime activation of GAL4 lines at 29°C compared to the 12 hr period occurring one day before activation. Outside of activation, flies were maintained at 21°C in a 12:12 LD cycle. Each GAL4 was compared to an enhancer-less GAL4 driver (pBDP-GAL4). *** $p = 0.0008$, **** $p<0.0001$ by ANOVA with Dunnett's multiple comparison test.

B. Rebound sleep during the 12 hr period immediately after activation of GAL4 lines. Each GAL4 was compared to an enhancer-less GAL4 driver (pBDP-GAL4). **** $p<0.0001$ by ANOVA with Dunnett's multiple comparison test.

bioRxiv preprint doi: https://doi.org/10.64898/2026.02.28.708752; this version posted March 3, 2026. The copyright holder for this preprint (which was not certified by peer review) is the author/funder, who has granted bioRxiv a license to display the preprint in perpetuity. It is made available under aCC-BY 4.0 International license.

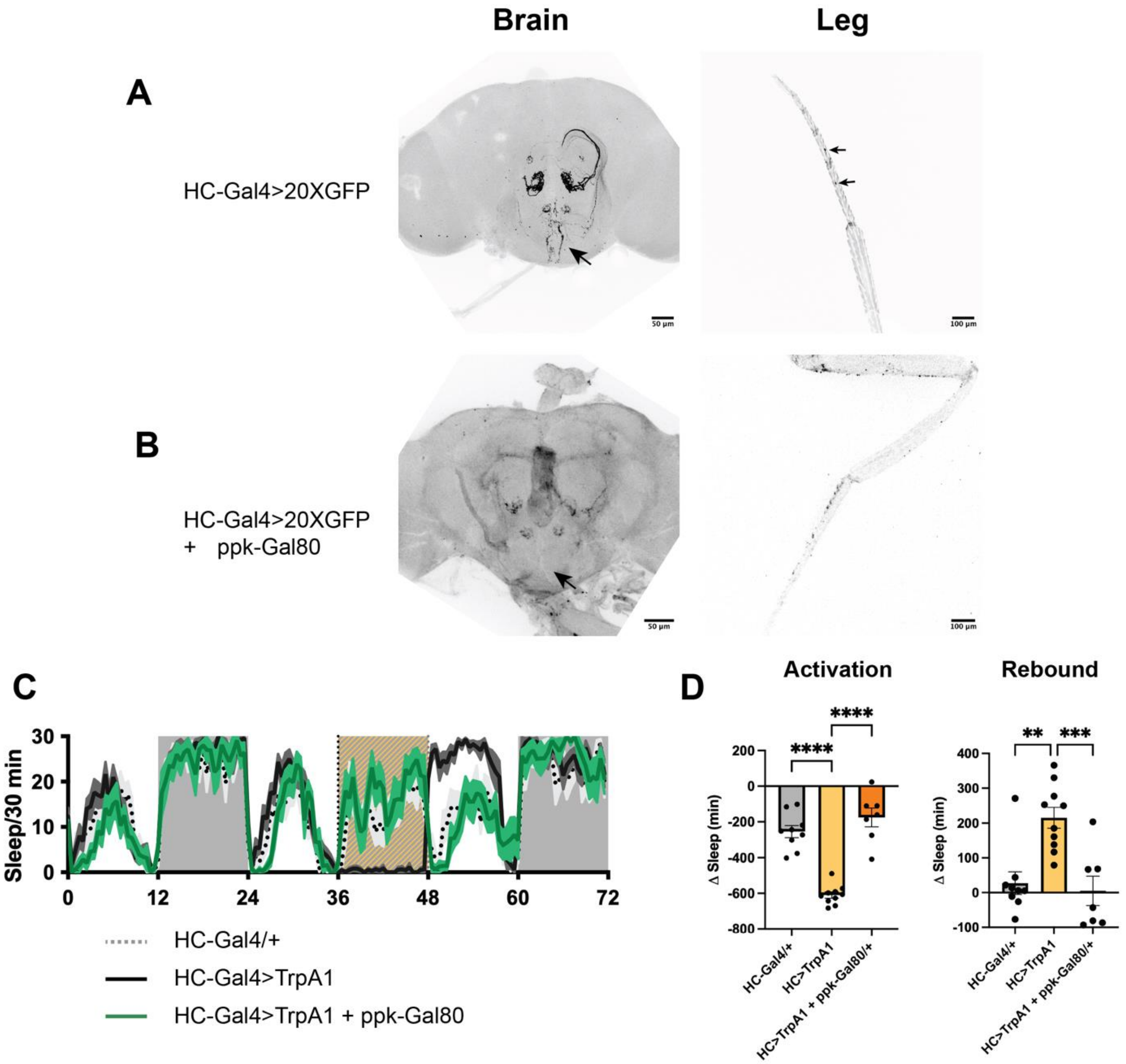

Figure S2. *pickpocket* leg neurons are necessary for HCGAL4 wake promotion and rebound.

Wake promotion and rebound in HC-GAL4 experiments is driven by ppk+ cells in the legs.

A. Confocal imaging of the brain and leg of flies in which HC-GAL4 was used to drive expression of GFP. GFP is shown in black. Arrow on the brain indicates terminals of the leg neurons in the subesophageal zone (SEZ) of the brain previously reported in Seidner et al. Arrows on the leg indicate two cell bodies associated with wake-promotion {Satterfield, 2022}.

B. Confocal imaging of the brain and leg of flies in which HC-GAL4 was used to drive expression of GFP in the presence of a ppk-Gal80 transgene blocking expression in ppk neurons. The absence of terminals in the SEZ is indicated by the arrow. Cell bodies in the distal part of the leg are also missing.

C. Coexpression of ppk-Gal80 completely blocked wake promotion during a 29°C nighttime pulse indicated in orange.

D. Quantification of the data in panel C indicates that coexpression of ppk-Gal80 abrogated the wake promoting effects observed when activating HC-GAL4 neurons with a TrpA1 transgene. **** $p<0.0001$ by ANOVA with Tukey's multiple comparisons.

E. Quantification of the data in panel C indicates that coexpression of ppk-Gal80 abrogated the wake promoting effects observed when activating HC-GAL4 neurons with a TrpA1 transgene. ** $p = 0.0013$, *** $p = 0.0008$ by ANOVA with Tukey's multiple comparisons.
